# CAR T Cell Toxicity: Current Management and Future Directions

**DOI:** 10.1097/HS9.0000000000000186

**Published:** 2019-03-29

**Authors:** Lucrecia Yáñez, Miriam Sánchez-Escamilla, Miguel-Angel Perales

**Affiliations:** 1 Department of Hematology, University Hospital Marqués de Valdecilla, Santander, Spain; 2 Department of Hematological Malignancies and Stem Cell Transplantation, Research Institute of Marques de Valdecilla (IDIVAL), Santander, Spain; 3 Department of Medicine, Adult Bone Marrow Transplant Service, Memorial Sloan Kettering Cancer Center, New York, NY; 4 Weill Cornell Medical College; New York, NY.

## Abstract

By late 2018, 2 chimeric antigen receptor T (CAR T) cell products have been approved by US and European regulatory authorities. Tisagenlecleucel (Kymriah, Novartis) is indicated in the treatment of patients up to 25 years of age with B-cell acute lymphoblastic leukemia (ALL) that is refractory or in second or later relapse, or adult patients with large B-cell lymphoma relapsed or refractory (r/r) after 2 or more lines of systemic therapy, including diffuse large B-cell lymphoma (DLBCL) not otherwise specified, high grade B-cell lymphoma and DLBCL arising from follicular lymphoma. Axicabtagene ciloleucel (Yescarta, Kite) is indicated for the treatment of adult patients with large B-cell lymphoma relapsed or refractory after 2 or more lines of systemic therapy, including DLBCL not otherwise specified, primary mediastinal large B-cell lymphoma, high grade B-cell lymphoma, and DLBCL arising from follicular lymphoma (ZUMA-1 trial).

This review will offer a practical guide for the recognition and management of the most important toxicities related to the use of the current commercial CAR T cells, and also highlight strategies to diminish these side effects in the future.

## Introduction

In 1965, Dr Thomas described some of the challenges facing the nascent field of allogeneic hematopoietic cell transplantation (HCT). At the time, clinical observations led to the knowledge that the use of immunosuppressive drugs and donor selection based on histocompatibility matching could reduce the incidence of marrow graft rejection and the incidence and severity of secondary disease, which we now know as graft-versus-host disease (GVHD).^1^ Fifty years later, we have made significant advances in our understanding of the pathophysiology of GVHD, and its prevention and treatment.^2–4^ Today, similar to the challenges faced by the pioneers of allogeneic HCT, we are living in the dawn of a new era of cellular therapies for malignant diseases based on the genetic modification of T cells and other lymphoid cells, and we are learning how to manage unexpected toxicities and their causes.

By late 2018, 2 chimeric antigen receptor T (CAR T) cell products have been approved by US and European regulatory authorities. Tisagenlecleucel (Kymriah, Novartis)^5^ is indicated in the treatment of patients up to 25 years of age with B-cell acute lymphoblastic leukemia (ALL) that is refractory or in second or later relapse (ELIANA trial),^6^ or adult patients with large B-cell lymphoma relapsed or refractory (r/r) after 2 or more lines of systemic therapy, including diffuse large B-cell lymphoma (DLBCL) not otherwise specified, high grade B-cell lymphoma and DLBCL arising from follicular lymphoma (JULIET trial).^7^ Axicabtagene ciloleucel (Yescarta, Kite/Gilead)^8^ is indicated for the treatment of adult patients with large B-cell lymphoma relapsed or refractory after 2 or more lines of systemic therapy, including DLBCL not otherwise specified, primary mediastinal large B-cell lymphoma, high grade B-cell lymphoma, and DLBCL arising from follicular lymphoma (ZUMA-1 trial).^9^ Additional approvals for products in the same indications as well as other malignant diseases such as myeloma are expected in the coming year.

This review will offer a practical guide for the recognition and management of the most important toxicities related to the use of the current commercial CAR T cells, and also highlight potential strategies to diminish these side effects in the future.

## Adverse effects of CAR T cell therapy

CAR T cells include a surface receptor that consists of a chimeric molecule composed of an extracellular domain derived from a B cell, that recognizes cell surface antigens, and which is linked to 1 or more intracellular T cell signaling domains via a transmembrane sequence.^10^ Although the most common toxicities are cytokine release syndrome (CRS) and CAR T cell-related encephalopathy syndrome (CRES),^10,11^ more recently termed immune effector cell-associated neurotoxicity syndrome (ICANS), other adverse events occur after CAR T cell infusion and need to be taken into consideration in clinical practice.

### Monitoring CAR T cell toxicity: clinical and laboratory work-up

Similar to the infusion of stem cell grafts and other cellular products, infusion of CAR T cell products is generally safe, but some precautions are needed. Pre-medication with acetaminophen and diphenhydramine should be administered 30 to 60 minutes before CAR T cell infusion.^5–9^ It is important to note that prophylactic use of systemic corticosteroids may interfere with the activity of the CAR T cells,^12^ and is not recommended. Vital signs (temperature, respiration rate, pulse, blood pressure, and oxygen saturation by pulse oximetry) are measured prior to, during and after the CAR T cell infusion in short time intervals.^7,13,14^ During the infusion and shortly thereafter, oxygen as well as emergency drugs and equipment should be readily available.^6,7,9^

After CAR T cells infusion, patients require close monitoring while they are at risk for the development of CRS or CRES.^13–15^ This observation period and the decision on inpatient versus outpatient monitoring are variable and depend on several factors. Inpatient monitoring should be indicated in those patients with high tumor burden because of their higher risk of CRS, neurotoxicity or tumor lysis syndrome (TLS).^13,16,17^ Patients with prior history of neurologic comorbidities are more likely to develop neurotoxicity^18^ and may also be considered for inpatient monitoring. There are also differences between the CAR T cell products infused. Whereas in the ZUMA trial, patients could be discharged at day 7 post treatment with axicabtagene ciloleucel in the absence of any sign of CRS or CRES,^9^ patients treated with tisagenlecleucel in the ELIANA and JULIET trial, had the option to be discharged same day after the CAR T cell infusion.^6,7^ Finally, a well instructed caregiver and an adequate infrastructure that allows outpatient visits and prompt access to emergency and intensive care units, with a specific location to manage these patients by staff trained in CAR T cell toxicities, are essential for outpatient management.^13–15^ Patients treated with tisagenlecleucel and axicabtagene ciloleucel should be instructed to remain within proximity (ie, 2 hours of travel) of a qualified clinical facility for at least 4 weeks following CAR T cell infusion.^5,8^

In Europe, the product information of tisagenlecleucel^5^ and axicabtagene ciloleucel^8^ specify that physicians should consider hospitalization for the first 10 days post infusion or at the first signs or symptoms of CRS and/or neurologic events. Patients should be monitored daily for the first 10 days following infusion of tisagenlecleucel and axicabtagene ciloleucel for signs and symptoms of CAR T cell related toxicities.^5,8^ After the first 10 days following the infusion, the patient should be monitored at the physician's discretion.^5,8^

For patients followed in an outpatient setting, temperature should be checked twice a day for at least the first 14 days after CAR T cell infusion,^6^ and preferably for 3 to 4 weeks.^5,8^ The patient and caregiver should be instructed to be alert to any symptom (back pain, skin rash, dizziness, chills, shortness of breath, chest pain, neurologic events…) or sign (tachycardia, hypotension) of CRS, Central Nervous System (CNS) toxicity or tumor lysis syndrome (TLS) for possible hospitalization.^13^

In patients who remain hospitalized after the CAR T cell infusion, vital signs should be assessed every 4 hours or more frequently if the patient experiences fever, hemodynamic changes, dyspnea and/or hypoxia (oxygen saturation <92% on room air) or neurologic symptoms.^14,19^ Fluid balance should be closely monitored, as well as daily weight.^14^ Assessment and grading of CRS should be done at least twice a day and whenever there are changes in patients’ status.^14^ Neurological evaluation to assess the CNS toxicity should include evaluation of mental status, headache and abnormal movements and be performed every 8 hours, or more frequently in the presence of changes.^14^

A complete blood count and biochemistry profiling, which includes basic metabolic panel, magnesium, phosphorus, uric acid and lactate dehydrogenase, liver enzymes, albumin and total bilirubin as well as coagulation tests with prothrombin time, partial thromboplastic time, fibrinogen, and D-dimer, C reactive protein and ferritin levels should be monitored daily in patients who are followed inpatient^13,14^ and when the visit is performed in the outpatient setting.^13^

## Cytokine release syndrome

CRS is the most frequent serious adverse event after CAR T cell therapy. Incidence of CRS in patients with ALL and NHL treated with tisagenlecleucel is 77%^6^ and 57%,^7^ respectively. In contrast, incidence of CRS in patients with NHL treated with axicabtagene ciloleucel is 93%.^9^ Differences in CRS incidence between both products are not exactly comparable because they were measured with different scales in the published studies. The CRS was graded according to the criteria of Porter et al in patients treated with tisagenlecleucel,^6,7^ whereas those treated with axicabtagene ciloleucel were graded with the Lee et al criteria.^9^

### Pathophysiology of CRS

The mechanism of CRS related to CAR T cell therapy can be divided in 2 main steps. First, the interaction between the CAR T cells and its target causes the activation and expansion of the CAR T cells and lysis of normal and tumor cells. This is associated with release of several cytokines such as interferon-γ (IFN-γ) and tumor-necrosis factor α (TNF-α).^20^ Second, the combination of these signals triggers the activation of monocytes and macrophages with enhanced tumoricidal capacity.^21^ The activated macrophages secrete high levels of pro-inflammatory cytokines (IL-6, IL-1, IL-10)^21^ and other mediators such us inducible nitric oxide synthase (iNOS),^22^ resulting in progression of CRS. In addition, the endothelium^23,24^ and myeloid cells^22^ also seem to be important mediators for CRS development and severity.

### Clinical presentation of CRS

Although CRS can occur up to 3 weeks after CAR T cell infusion, the median time to onset of CRS is 2 days (range 1–12 days) for axicabtagene ciloleucel^9^ and 3 days for tisagenlecleucel (range 1–22 days in ALL patients and up to 9 days in NHL patients).^6,25^ Clinically, CRS can present with a variety of symptoms ranging from a prodromal syndrome to life-threatening manifestations. The prodromal syndrome of CRS includes a flu-like syndrome with fever, fatigue, headache, arthralgia, myalgia, and malaise. Pyrexia (fever > 38°C) is the most frequent, and usually the first, clinical sign of CRS. In some cases, it rises above 40°C and, compared to patients with mild or moderate CRS, fever in patients with sCRS peaks earlier and has a longer duration.^23^ Gastrointestinal symptoms such as nausea, diarrhea and vomiting, are also common.^5,8^ Severe CRS, characterized by hemodynamic instability and organ dysfunction, is often preceded by mild or moderate signs such as hypoxia and mild hypotension, so clinicians should be alert.

### Current management of CRS

**Figure 1:**
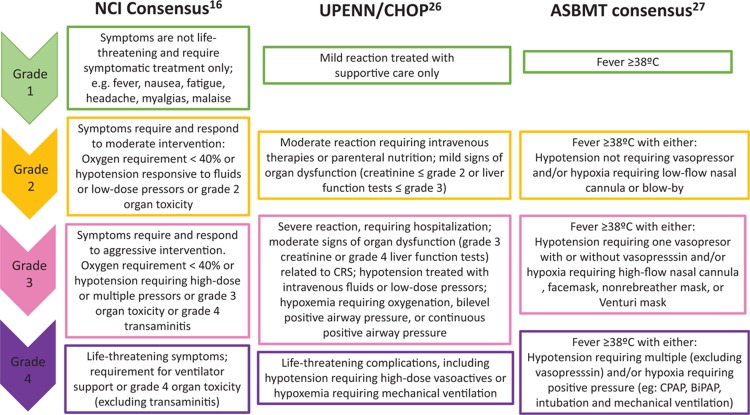
**The most commonly used grading system for monitoring and treatment of CRS after CAR T cells**. CRS = cytokine release syndrome, CAR T = chimeric antigen receptor T.

The current management of CRS follows a grading system based on vital signs and symptoms. The grading systems most commonly used for the monitoring and treatment of CRS after CAR T cells are the NCI and PENN/CHOP grading systems.^16,26^ While the both include a four level scale of severity, there are some differences. To resolve these discrepancies, the American Society of Blood and Marrow Transplantation (ASBMT) convened a consensus conference in June 2018 to develop a common grading system for both CRS and neurotoxicity.^27^ In this consensus, CRS grading is driven by hypotension and/or hypoxia and CRS grade is determined by the more severe event (Fig. 1).

When symptoms of early CRS appear, patients should be under close observation and provided with symptomatic support with antipyretics and/or analgesics trying to avoid NSAIDs, which can affect renal function.^19^ In addition, infection should be excluded in febrile patients and empiric antibiotics administered if the patient is neutropenic, given the increased risk of infection and prior lymphodepletion regimen. In a recent article, one of every four patients who received CD19-targeted CAR T cell therapy experienced an infection, mainly bacteremias and respiratory viral infections, within the first 4 weeks after the CAR T infusion.^28^ The use of prophylactic antibiotics has not been established, though they are used at some centers. Additional CRS side effects can be managed with antiemetics, oxygen, intravenous fluids and/or low-dose vasopressors as needed, while avoiding the use of corticosteroids.^14,15^

Severe CRS (sCRS), defined as ≥ Grade 3 by Penn grading system for tisagenlecleucel or Lee grading system for axicabtagene ciloleucel, occurred in 46% of patients with relapsed/refractory B-ALL treated with tisagenlecleucel,^6^ and 13% to 18% of patients with relapsed/refractory DLBCL treated with axicabtagene ciloleucel^9^ and tisagenlecleucel,^7^ respectively. In one study, patients who developed severe CRS tended to have earlier onset of symptoms.^23^ The prompt recognition of sCRS and rapid institution of treatment are critical. Similar to septic shock, patients exhibit hemodynamic instability despite intravenous fluids and vasopressor support, worsening respiratory distress, including pulmonary infiltrates, and increasing oxygen requirement that can include the need for high-flow oxygen and/or mechanical ventilation, and rapid clinical deterioration associated with liver and renal dysfunction. This multi-organ system failure requires intensive medical management and the majority cases responses to IL-6 blockade with tocilizumab.^10,16,19,29^

**Figure 2:**
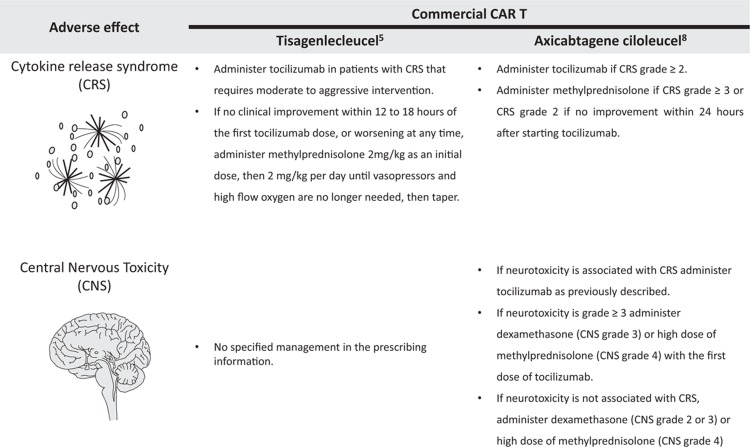
**Management of CRS and neurotoxicity based on prescribing information of tisagenlecleucel and axicabtagene ciloleucel**. CRS = cytokine release syndrome.

Tocilizumab (Roche) is an IL-6 receptor antagonist that was approved by the FDA for the management of severe CRS and has demonstrated a high response rate in patients with sCRS.^6,7,9,29,30^ Siltuximab (Janssen) binds to soluble IL-6, but has not been studied as first-line therapy for CRS and is not currently approved for this indication. Although the clinical status that triggers the recommendation to start tocilizumab is variable between the two commercial CAR T cell products^5,8^ (Fig. 2), the median time from the beginning of CRS to the first dose of tocilizumab is similar between tisagenlecleucel and axicabtagene ciloleucel (3 and 4 days, respectively).^29^ If there is no clinical improvement, tocilizumab is given as needed at a minimum interval of 8 hours to a maximum total of 4 tocilizumab doses.^5,8^ In general, the resolution of symptoms is achieved within the first days after the start of tocilizumab, often within a few hours, and nearly all patients require one or two doses.^20,29^ In the absence of clinical improvement within 12 to 24 hours after starting tocilizumab, or in the presence of worsening at any time, corticosteroids are administered and tapered over 3 days.^5,8^ Fortunately, only a small number of patients will develop resistant CRS in which neither tocilizumab nor corticosteroids are effective.^17,20^ This situation is related with a very high mortality^17^ and other therapies that interfere with inflammatory cytokine pathways such as anti-TNFα (etanercept) or IL-1R inhibitor (anakinra) should be considered.^16,22,31^

The median time to CRS resolution ranges between 7 days in patients treated at the JULIET trial^24^ and 8 days for those treated at the ELIANA and ZUMA trial.^6,9^ However, additional complications may result from CRS and its treatment. The severity of CRS was the only factor associated with infection in a multivariable model that also included the presence of ANC <0.5 × 10^9^/L on the day of infection, the maximum neurotoxicity grade, treatment with tocilizumab and/or corticosteroids, and ICU admission. In addition, patients with severe CRS present prolonged cytopenia and develop more frequent invasive mold infections,^28^ so broad spectrum antifungal prophylaxis in this group of patients should be strongly considered.

## Central Nervous System (CNS) toxicity

The incidence of CNS toxicity ranges from 0 to 87% and seems to be most frequent in immature B cell diseases.^6,7,9,33,34^ In fewer than 10% of patients, the onset of CNS toxicity occurs in the absence of CRS. However, in these patients, neurologic signs and symptoms are typically mild (grade 1). In the other 90% of patients, CNS toxicity appears concurrent with CRS or following its resolution.^18,34^

### Pathophysiology of CNS toxicity

**Figure 3:**
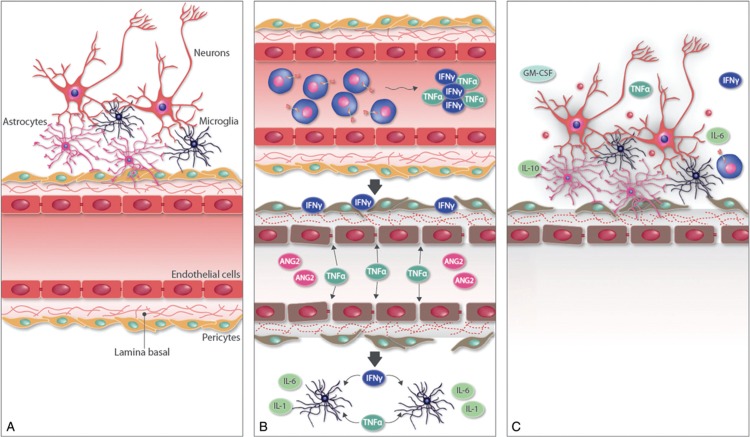
**Model of neurotoxicity secondary to CAR T cell therapy adapted from Gust et al**^18^**and Santomasso et al**.^34^ A) Components of the blood-brain-barrier. B) CAR T cell activation and expansion produces the release of TNF-α and IFN-γ. TNF-α together with other molecules that can be present even before the CAR T cell infusion, such as ANG2, are able to activate the endothelial cells and disrupt the joints between them. In addition, pericytes exposed to IFN-γ contribute to endothelial cells activation and increase BBB permeability. After the disruption of the blood-brain-barrier IFN-γ and TNF-α can activate the microglia. C) The cytokines released upon the activation of the microglia induces an inflammatory state and brain damage. CAR T = chimeric antigen receptor T.

Although the pathogenesis of neurotoxicity is not fully understood and was first thought to be related to direct parenchymal CAR T cells toxicity,^35^ recent studies^18,34,36^ suggest that the dysfunction of the blood brain barrier (BBB) is the main factor. The BBB is formed by capillary endothelial cells surrounded by extracellular matrix (basal lamina), pericytes, microglia and astrocytes.^37^ In addition to other factors,^37^ impairment of the BBB function has been related to TNF-α, IL-6 and IL-1,^37,38^ and the angiotensin 1 (ANG1) and angiotensin 2 (ANG2) balance.^39,40^ All these factors, as well as other molecules implicated in the expansion and activation of the CAR T cells, myeloid cells, monocyte and macrophages (IFN-γ, IL-10, G-CSF, GM-CSF, IL-8, MCP-1),^18,34,36^ and neurotoxic substances such as glutamate and quinolinic acid,^34^ have been found to be elevated in severe forms of CAR T cells neurotoxicity.^18,34,36^Figure 3 shows the plausible model of BBB dysfunction associated with CAR T cells therapy.

### Clinical presentation of CNS toxicity

The clinical features of neurotoxicity associated with CAR T are numerous and may vary from headache, pain, memory loss, meningismus, dizziness, alterations in mental status (somnolence, disorientation, impaired attention, agitation, delirium, coma), movement disorders (tremor, myoclonus, facial automatisms), impaired speech (dysartria, aphasia), seizures and encephalopathy to coma.^6,7,9,18,34^ When performed, electroencephalography shows a diffuse slowing in 76% of patients, or can detect clinical or subclinical seizures.^18,34^ Neuroimaging studies with MRI are usually normal except for those patients who develop severe CNS toxicity.^18,34^ In approximately 30% of these patients a reversible common pattern of T2/FLAIR hyperintensities affecting different regions is seen.^18,34^

Neurologic toxicities reported after the treatment with tisagenlecleucel for ALL^6^ and NHL^7^ or axicabtagene ciloleucel^9^ occurred within the first 8 weeks is 40%, 39%, and 64%, respectively. The median time to onset ranges from 4 to 6 days and the median duration of neurologic toxicities was 17 days for axicabtagene ciloleucel, and 6 and 14 days for patients for patients with r/r B-ALL and r/r DLBCL treated with tisagenlecleucel, respectively. In a recent study of r/r B-ALL patients treated with CD19-specific 19 to 28z CAR T cells in a phase I clinical trial (NCT0144069), the median duration of neurotoxicity was the same for patients who developed mild and/or severe CNS toxicity, 11 days.^34^

### Current management of CNS toxicity

Similar to CRS management, CNS toxicity needs to be promptly recognized. There is no consensus on the use of seizure prophylaxis with levetiracetam in CAR T cell patients. Whereas some centers^14^ prescribe it prior and up to day +30 of CAR T cell infusion in all patients, others^15^ limit prophylactic levetiracetam to patients with high risk of CNS toxicity (ie, prior history of CNS toxicity, CNS co-morbidity, or CNS leukemia) or in patients who develop neurotoxicity. Patients who experience neurotoxicity should be monitored closely.^14^ Transfer to the ICU is recommended for patients grade ≥3 CNS toxicity^15^ and can be considered in patients with grade 2 toxicity according to the center's policy.^14^ In some cases, neurologic toxicity may also necessitate intubation and mechanical ventilation for airway protection in the absence of respiratory failure.^19^ In one trial (NCT01865617), fever ≥ 38.9°C occurring within the first 36 hours after the infusion of CD19 CAR-T cells containing a 4–1BB costimulatory domain^41^ had a 100% sensitivity of subsequent grade ≥4 neurotoxicity.^18^ The management of CNS toxicity is based on the use of corticosteroids, which are given at different doses depending on the severity, centers policy and the commercial product^5,8,14,15^ (Fig. 2). It is important to note that although tocilizumab is effective in CRS, neurotoxicity does not respond to anti IL-6 blockade in most patients and tocilizumab potentially may make it worse.^18,34,36,42^ The resolution of neurotoxicity seems to be longer than the time to resolution of CRS.^34^

## Other toxicities associated with CAR T cells

### Infusion reactions

CAR T cells are infused according to the manufacturer's instructions.^5,8^ Following these recommendations, infusion reactions are infrequent, generally mild, and usually occur during or immediately following the CAR T cell administration. The most common side effects are upper digestive symptoms (nausea and vomiting) and hypotension, attributable to the dimethyl sulfoxide (DMSO) cryoprotectant and to diphenhydramine pre-medication, respectively.^43^ However, similar to what can be observed with other cryopreserved products,^23^ anaphylaxis and severe infusion reactions can be observed, mainly related with the DMSO, dextran^5^ or residual compounds such as gentamycin.^8^ Tisagenlecleucel contains 7.5% DMSO,^5^ while axicabtagene ciloleucel^8^ contains 5% DMSO. It is unknown if DMSO can affect CAR T cell proliferation.^44^

### Tumor lysis syndrome

In contrast to other novel therapies for hematologic malignancies that have increased the incidence of tumor lysis syndrome (TLS),^45,46^ TLS after CAR T cell therapy is uncommon even in high risk situations.^5,8^ However, precautions such as intravenous hydration and prophylactic allopurinol or febuxostat should be administered prior to the initiation of conditioning lymphodepleting chemotherapy in those patients with elevated uric acid or high tumor burden.^6,7,9^ Signs and symptoms of TLS should be monitored and managed according to standard guidelines.

### Cytopenias

Cytopenias are the most common adverse effect of grade ≥3 after axicabtagene ciloleucel^9^ and tisagenlecleucel,^6,7^ and can be present for several weeks following the CAR T cells infusion.^6^ The most important factors related to the development of cytopenias include the conditioning regimen, cytokines released in CRS, the macrophage activation syndrome, and the exposure multiple prior chemotherapy treatments.^14,16,19^ Recently, a report from the Fred Hutchinson Cancer Research Center^47^ has shown that 20% of patients with CLL or NHL treated in a phase I/II Study of defined subsets of CD19 CAR T cells (NCT01865617) experienced ongoing cytopenias beyond the 3rd month after CAR T cell infusion, which required G-CSF and/or blood transfusions. In addition, and with a median follow-up of 23 months, 5% were diagnosed with myelodysplastic syndrome.

Neutropenia is the most common cytopenia.^6,7,9^ Patients with large B-cell lymphoma treated on the ZUMA-1 trial^9^ or JULIET trial,^7^ developed an absolute neutrophil count below 1.0 × 10^9^/L in 78% and 64% of cases, respectively. Similar results were found in children and young adult patients treated on the ELIANA trial in which 53% of patients had neutropenia grade ≥3 by day 28 after CAR T cells infusion. It is important to note that, although febrile neutropenia was observed in 36% of patients treated with axicabtagene ciloleucel^9^ and 17% to 37% with tisagenlecleucel,^6,7^ myeloid growth factors, particularly GM-CSF, are not recommended during the first 3 weeks after CAR T cell infusion or until CRS has resolved.^5^ Levels of G-CSF and GM-CSF have been found to be elevated in patients with severe neurotoxicity and may be related to its development.^34^ In addition, GM-CSF inhibition with the monoclonal antibody lenzilumab in xenograft model reduces CRS and neuroinflammation without diminishing the CAR T cell antitumor activity.^48^

Severe thrombocytopenia is also common with axicabtagene ciloleucel^9^ and tisagenlecleucel.^6,7^ Thrombocytopenia was observed in 38% of patients treated on the ZUMA-1 trial,^9^ in 11% of patients treated on the JULIET trial.^7^ It should be noted that the dose of cyclophosphamide was higher on the ZUMA-1 trial and this may be a contributing factor. Thrombocytopenia was unresolved by day 28 in 41% patients in the ELIANA trial.^6^ There is currently no information regarding the use or safety of thrombopoietin agonists in this subset of patients.

### Cardiac toxicity

The incidence of cardiac events (sinus tachycardia, arrythmias, cardiomyopathy and cardiac arrest) reported in the three clinical trials^6,7,9^ on which tisagenlecleucel or axicabtagene ciloleucel were approved, ranged from 29% to 39%. It is important to note that previous cardiac dysfunction or arrythmias are not a contraindication for CAR T cell therapy.^5,8^

To date, only one study performed in pediatric patients treated for B-ALL,^49^ has reported on the impact of prior cardiac disease on CAR T cell infusion, risk factors for cardiac dysfunction after treatment and the follow-up of these patients. In this study, high disease burden (blasts > 25% on bone marrow biopsy) was significantly associated with increased risk for cardiac events (p < 0.001). In addition, patients with lower ejection fraction or diastolic dysfunction before treatment required more frequently vasoactive drugs.^49^ At the time of discharge, follow-up echocardiograms showed that only 7% of patients had new systolic or diastolic dysfunction. Patients with cardiac dysfunction at the time of discharge were treated in an outpatient setting with ACE inhibitors or beta-blockers and were followed by cardiologists. Interestingly, the majority of patients had recovered from their cardiac dysfunction related to CAR T cells after 6 months of follow-up.^49^

### Hypogammaglobulinemia

Hypogammaglobulinemia is a delayed side effect of tisagenlecleucel and axicabtagene ciloleucel secondary to the persistence of the CAR T and subsequent development of B-cell aplasia.^16,19,26,50,51^ It seems to be higher in patients with r/r B-ALL treated with tisagenlecleucel (43%) compared to patients with r/r DLBCL treated with tisagenlecleucel (14%) or axicabtagene ciloleucel (15%). The presence of hypogammaglobulinemia is mainly associated with the achievement of a complete response.^6,7,9,50,52^ IgG levels typically fall 1 to 3 months after CAR T infusion and can remain low up to 4 years,^52,53^ but some patients can maintain antibody-secreting memory plasma cells contributing to long-lasting humoral immunity.^54^

There are differences in the management of hypogammaglobulinemia between children and adults. While replacement is typically done in pediatric patients,^6,52^ adult centers reserve the administration of intravenous immunoglobulin (IVIG) in those patients with severe or recurrent infections.^7,9^ The recommended dose of IVIG replacement for primary immunodeficiency is 400 to 600 mg/kg every 3 to 4 weeks.^55^ Replacement treatment with IVIG should to be maintained until IgG levels ≥ 400 mg/dl.^53^

The safety of immunization with live viral vaccines during or following tisagenlecleucel or axicabtagene ciloleucel treatment has not been studied. With both treatments, vaccination with live virus vaccines is not recommended for at least 6 weeks prior to the start of lymphodepleting chemotherapy and until immune recovery following treatment with CAR T.^5,8^

### Graft-versus-host-disease

Tisagenlecleucel and axicabtagene ciloleucel are made from T cells harvested from the recipient. In patients who have received a previous allogeneic stem cell transplantation, however, the T cells can be of donor origin. In the early phase trials with tisagenlecleucel (NCT01626495 and NCT01029366), 18 patients had received a previous allogeneic stem cell transplantation.^56^ Although the median donor chimerism at the time of leukapheresis was 100%, no patient developed graft-versus-host disease after the CAR T cells infusion.^56^ Interestingly, absence of new onset acute graft-versus-host disease has also been reported when the CAR T cell were provided by the patient's transplant donor in a phase I clinical trial (NCT01087294).^57^

## Future strategies to mitigate toxicities related with CAR T cell therapy

**Figure 4:**
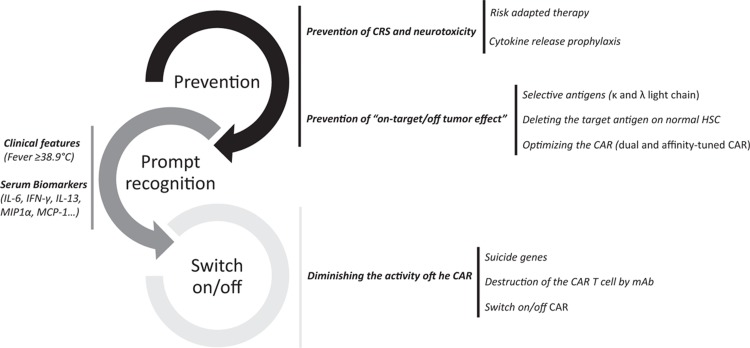
**Future strategies to decrease side effects related with CAR T cell therapy**. CAR T = chimeric antigen receptor T.

Many of the following strategies are in investigational and should not be considered as standard of care. With these caveats in mind, the strategies to decrease the number and intensity of the most frequent and/or severe side effects related to CAR T cell therapy can be divided into prevention of CRS and neurotoxicity, prompt recognition with clinical and/or biological predictive models and, when the side effect appears, diminishing the activity of the CAR T cells (Fig. 4).

## Prevention of CRS and neurotoxicity

The risk for CRS and/or neurotoxicity is mainly related to the disease burden, the CAR T cell dose infused and recipient factors such as age and preexisting neurologic comorbidities.^18,20,24^ The adjustment of the treatment based on the patient's risk, and the administration of several drugs to avoid the “cytokine storm” are strategies to prevent both effects.

### Risk adapted therapy

The burden of CD19+ B cells in the marrow for ALL or lymph nodes in NHL is an important risk factor for CRS and neurotoxicity development.^10^ There is not a standardized number of bone marrow leukemic blasts (5–50%) or a maximum diameter of lymph node size (5–10 cm) that is considered bulky disease in CAR T cell therapy.^5,7–9,24,58,59^ In B-ALL, bone marrow infiltration >20% has led some investigators to reduce the recommended CAR T dose from 2 × 10^6^ cell/kg to 2 × 10^5^ cell/kg;^59^ and in patients with DLBCL, 2 × 10^6^ cell/kg seems to be the maximum tolerated dose.^59^ In addition, a reduction of the CAR T cell dose might be considered in patients with preexisting endothelial damage,^18^ because of its role in the development of CRS and neurotoxicity. It should be noted that, according to the package inserts, a single dose of axicabtagene ciloleucel contains 2 × 10^6^ cell/kg, but the number of CAR T cells that can be administered of tisagenlecleucel can range between 0.6 to 6.0 × 10^8^ cell/kg.^5,8^ In addition, if possible, debulking therapy to decrease the disease burden should be considered.^60^

### “Cytokine release prophylaxis”

Locke et al,^42^ has reported that the use of tocilizumab, started at day 2 after CAR T cell infusion, may reduce the incidence of severe CRS (less than 5%) but not grade ≥ 3 CNS toxicity in patients with NHL treated with axicabtagene ciloleucel. Ibrutinib, a first generation BTK inhibitor, has been studied in the setting of CAR T cell therapy.^61,62^ It may enhance the generation of CAR T cells in patients with CLL and can improve engraftment of CD19 CAR T cells in a murine model.^62^ Similarly, in a xenograft model of CRS, Ibrutinib is capable of reducing the production of inflammatory cytokines, especially IFN-γ, from both CAR T and tumor cells, thus diminishing the intensity of CRS.^61^ The blockade of the IL-1 receptor, as well as GM-CSF inhibition, are emerging as interesting therapeutic targets for the prevention of CRS and neurotoxicity. The pharmacologic blockade of the IL-1 receptor with anakinra or the construction of a CAR T cell capable of producing its own IL-1 receptor antagonist, and GM-CSF neutralization with lenzilumab have demonstrated reduction of both CRS and neurotoxicity in xenograft models, without compromising antitumor efficacy.^22,48,63^ Finally, endothelium has acquired an important role in the development of CAR T cell toxicity, especially for neurotoxicity, and this remains an active area of investigation.^18^

## Prompt recognition of severe CRS and neurotoxicity: predictive biomarkers

The best predictive biomarker must be able to predict early, ideally in the first 24 to 36 hours after the CAR T cell infusion, the onset of severe CRS or neurotoxicity with a high sensitivity and specificity, and it should also be available in most clinical settings.^24^

Ferritin and C reactive protein (CRP) were thought to be useful markers for prediction of severe CRS (grade ≥4). In a study on B-ALL patients,^64^ a peak ferritin >10,000 mg/d was detected in all patients with severe CRS and early C reactive protein (CRP) elevation was also associated with grade 4 to 5 CRS. However, a CRP >6.8 mg/dl would have identified only 72% of the high-risk CRS cases and had a low positive predictive value (43%). Neither ferritin nor CRP, nor other biochemical parameters like LDH, AST, ALT, BUN, and creatinine were found to predict the severity of CRS and neurotoxicity.^16,64^ Recently, elevated levels (≥1343.5 pg/ml) of monocyte chemoattractant protein-1a (MCP-1) in serum of B-ALL, NHL and CLL patients, combined with the presence of fever ≥38.9°C within 36 hours of CAR T cell infusion has shown the capacity of identify patients who develop grade ≥4 CRS with 100% of sensitivity and 95% of specificity.^23^

For neurotoxicity, MSKCC and FHRC groups^18,34^ have demonstrated how preexisting markers of endothelial activation, like the angiopoietin axis seems to be important in the development of severe neurotoxicity, however they have not been yet investigated as predictive biomarkers. Table 1 shows four predictive models to detect early CRS or CNS toxicity, but all require further validation studies.

**Table 1:**
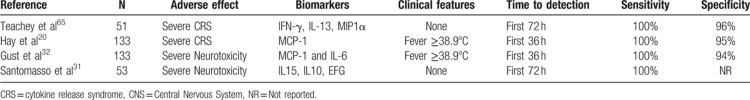
Predictive models to detect early CRS or CNS toxicity.

## Prevention of “on-target/off tumor effect”

As noted above, the persistence of CD19+ CAR T cells can cure patients of their B lymphoid malignancy but is associated with a long-term B cell aplasia.^6,51,52^ To diminish these undesirable side effects, several strategies can be considered.

### Selective antigens

Targeting a clonally restricted B cell marker, such as the κ and λ light chain of immunoglobulins, maintains antitumor activity without compromising humoral immunity.^65,66^ This approach can be feasible in mature B cell malignancies, but it cannot be extended to B-ALL because of the lack of expression of this antigen in immature cells.

### Deleting the target antigen on normal hematopoietic stem cells

Kim et al,^67^ recently published a model, first in mice and subsequently in rhesus macaques, that combines the infusion of previously genetically modified CD33-deficient stem cells and the infusion of CD33+ CAR T cells targeting AML. In this study, the authors demonstrate a long-term multilineage engraftment of gene-edited cells with normal myeloid function.

### Optimizing the CAR

Engineering a dual CAR that simultaneously recognizes 2 or more tumor specific antigens or an affinity-tuned CAR that acts against antigens based on the intensity of expression may result in higher accuracy in the recognition of target and diminish the probability of relapse due to target antigen deletion.^68,69^

## Diminishing the activity of the CAR

When severe toxicity appears, besides the use of drugs such as tocilizumab or corticosteroids, it would be highly desirable to be able to temporarily switch off the CAR T cells. Currently, there are significant efforts in the development of control systems for CAR T cells.^68^ These approaches are based on the introduction of suicide genes into CAR T cells using inducible caspase-9 or herpes virus thymidine kinase, co-expression of a protein that can be recognized by approved monoclonal antibodies, such as rituximab or cetuximab, the introduction of a small molecule that binds to the antigen and CAR T cell and is broken down in a few hours, or by using the CRISPR/Cas9 genome editing technology, which is able to modify the expression of the CAR on the surface of the T cell.^68–71^

## Conclusion

The benefit of CAR T cells has been demonstrated in relapsed/refractory B cell malignancies that express the CD19 antigen.^6,7,9^ A detailed understanding of the early and late toxicities associated with this therapy and their management is essential for the safe use of the recently FDA and EMA approved CAR T cells, tisagenlecleucel and axicabtagene ciloleucel. Moreover, strategies to diminish the toxicity based on prevention and prompt recognition of severe adverse events are currently available for clinical use. Furthermore, ongoing development of new generation CAR T cells provide the opportunity to increase the cure rate of CAR T cells, while decreasing their toxicity.
